# Self‐Perceived Confidence in Pediatric Dental Procedures Among Final‐Year Dental Students

**DOI:** 10.1111/ipd.70142

**Published:** 2026-09-11

**Authors:** Ceren Kaplan, Melis Ünsal Çalışkaner

**Affiliations:** ^1^ Department of Pediatric Dentistry, Faculty of Dentistry Istanbul Altınbaş University Istanbul Turkey; ^2^ Department of Pediatric Dentistry, Faculty of Dentistry Istanbul Atlas University Istanbul Turkey

**Keywords:** dental students, pediatric dentistry, self‐perceived confidence, undergraduate dental education

## Abstract

**Purpose/Objectives:**

This study aimed to assess the self‐perceived confidence of final‐year dental students in performing pediatric dental procedures. The study included students who had completed their pediatric dentistry internship.

**Methods:**

A descriptive, cross‐sectional survey was conducted among final‐year dental students (*n* = 46) at Istanbul Atlas University during the 2024–2025 academic year. Data were collected using a self‐administered 15‐item Likert‐scale questionnaire assessing students' self‐perceived confidence in performing pediatric dental procedures, including preventive and more technically demanding clinical interventions.

**Results:**

Students reported high levels of self‐perceived confidence in basic pediatric dental procedures, such as fissure sealant application and oral hygiene education. Lower confidence was observed for more technically demanding procedures, including pulpotomy and stainless steel crown placement. Exploratory analyses initially identified gender differences in three questionnaire items; however, these differences were no longer statistically significant after adjustment for multiple comparisons.

**Conclusion:**

Final‐year dental students reported high self‐perceived confidence in routine pediatric dental procedures but lower confidence in technically demanding procedures. These findings may help identify areas where additional clinical experience and educational support could further enhance students' self‐perceived confidence in pediatric dentistry.

## Why This Paper Is Important to Pediatric Dentists

- This study provides insight into final‐year dental students' self‐perceived confidence in performing pediatric dental procedures.
- Students reported higher self‐perceived confidence in routine pediatric dental procedures. Lower confidence was observed for more technically demanding procedures, particularly stainless steel crown placement and pulpotomy.
- These findings may help pediatric dental educators identify areas for curriculum review and inform future evaluations of undergraduate pediatric dentistry education.

## 1 Introduction

Pediatric dentistry is a multifaceted discipline that requires not only technical proficiency in dental procedures but also communication skills tailored to children's developmental stages, effective behavior management strategies, emotional regulation, and self‐confidence in managing pediatric patients [1, 2]. Children differ significantly from adults in terms of cognitive maturity, fear levels, and behavioral responses to the clinical environment. Consequently, providing dental care for pediatric patients presents unique challenges, particularly for dental students who are still developing their clinical skills and confidence [3]. Previous studies have shown that students perceive pediatric dentistry as one of the most stressful and challenging clinical specialties. This perception stems from the combination of psychosocial and technical challenges involved in treating young patients [4, 5].

Among the primary sources of stress experienced by dental students in pediatric dentistry are uncooperative child behaviors, parental pressure or interference, perceived difficulties in pain and local anesthesia management, and challenges in applying behavior guidance techniques [6]. Although behavior management strategies such as tell–show–do, distraction, and positive reinforcement are well established theoretically, their implementation in clinical practice remains challenging for many students [7]. Furthermore, students consistently report lower self‐perceived confidence and higher anxiety when performing more complex pediatric procedures, including local anesthesia, pulp therapy, and dental trauma management [5, 8].

Previous studies have shown that self‐perceived confidence is an important educational outcome in undergraduate dental education, as it may influence students' clinical decision‐making, willingness to perform clinical procedures, and preparedness for clinical practice [9]. Structured clinical training, adequate exposure to a wide range of pediatric cases, faculty supervision, and hands‐on learning opportunities are considered essential components of undergraduate pediatric dental education. These educational experiences have been associated with higher levels of self‐perceived confidence among dental students in pediatric dentistry [10]. However, most published studies have focused on general dental student populations or mixed cohorts, whereas evidence specifically evaluating final‐year dental students following completion of their pediatric dentistry internship remains limited [11, 12].

Although pediatric dentistry education varies across countries, many studies have reported that undergraduate dental students often feel insufficiently prepared to manage pediatric patients, particularly during more complex clinical procedures [13, 14]. However, recent evidence from Turkey remains limited [15, 16]. Few studies have specifically evaluated the self‐perceived confidence of final‐year dental students after completing their pediatric dentistry internship. This lack of evidence makes it difficult to evaluate current undergraduate training and identify areas requiring further educational support. As competency‐based dental education continues to evolve, further research is needed to better understand students' self‐perceived confidence at the completion of their undergraduate training [17].

Assessing the self‐perceived confidence of final‐year dental students following completion of their pediatric dentistry internship is important for understanding how confident they feel in performing common pediatric dental procedures. Particular attention should be given to procedures such as local anesthesia, pulp therapy, dental trauma management, and behavior guidance, which are generally perceived as more challenging during undergraduate training. Evaluating students' self‐perceived confidence in these areas may provide valuable insight into the effectiveness of current pediatric dentistry training and help identify aspects of clinical education that could benefit from further support. Therefore, this study aimed to assess final‐year dental students' self‐perceived confidence in performing pediatric dental procedures after completing their pediatric dentistry internship.

## 2 Materials and Methods

This descriptive, cross‐sectional questionnaire‐based study was conducted during the 2024–2025 academic year to assess the self‐perceived confidence of final‐year dental students in performing pediatric dental procedures following completion of their pediatric dentistry internship. The study was reported in accordance with the Strengthening the Reporting of Observational Studies in Epidemiology (STROBE) statement for cross‐sectional studies. It was conducted at the Faculty of Dentistry, Istanbul Atlas University, Turkey, and included final‐year undergraduate dental students. Data were collected using a self‐administered questionnaire consisting of two sections: 2 demographic questions (gender and age) and 15 items assessing students' self‐perceived confidence in performing pediatric dental procedures. Ethical approval was obtained from the Non‐Interventional Scientific Research Ethics Committee of Istanbul Atlas University (Approval No. 05/16, May 26, 2025) before the commencement of participant recruitment and data collection. The study was conducted in accordance with the Declaration of Helsinki. Written informed consent was obtained from all participants prior to participation, and all study procedures, including participant recruitment and data collection, were conducted only after ethical approval had been granted.

### 2.1 Study Participants

Participants were recruited from the population of final‐year undergraduate dental students at the Faculty of Dentistry, XX XX University, Turkey, during the 2024–2025 academic year. The inclusion criteria were students who were enrolled in the final year of the undergraduate dental program, had successfully completed all theoretical courses and clinical internship requirements in pediatric dentistry, and provided informed consent to participate in the study. Students who had not completed the pediatric dentistry curriculum, were not enrolled in the final year of the undergraduate dental program during the 2024–2025 academic year, or declined participation were excluded from the study. All eligible students (*n* = 46) were invited to participate through an online questionnaire generated using Google Forms (Google LLC, Mountain View, CA, USA). As all eligible final‐year dental students were invited to participate, no formal sample size calculation was performed, and the study therefore represents a census of the eligible study population. The questionnaire link was distributed through the institutional email system by the faculty administrative office before graduation. Participation was voluntary, no incentives were offered, and responses were collected anonymously to ensure participant confidentiality. All invited students completed the questionnaire, resulting in a response rate of 100%. All questionnaire items were mandatory in Google Forms; therefore, all submitted questionnaires were complete, and no missing data were recorded or required handling.

To minimize potential sources of bias, all eligible final‐year students were invited to participate, participation was voluntary, responses were collected anonymously, and all questionnaire items were mandatory, thereby reducing selection bias and missing data.

### 2.2 Survey Tool

At the study institution, undergraduate students receive preclinical pediatric dentistry simulation training during the third year of the dental curriculum. Clinical pediatric dentistry training is delivered through two 4‐week clinical rotations in the fourth and fifth years. During these supervised clinical internships, students are required to complete a predefined minimum number of core pediatric dental procedures, including preventive, restorative, pulp therapy, stainless steel crown, and extraction procedures, before successfully completing the rotation. Student performance is evaluated based on the completion of these clinical requirements under faculty supervision.

A self‐administered questionnaire was developed by adapting relevant items from the questionnaires developed by Hossain et al. and Rodd et al. [13, 18] The original items were reviewed and adapted to reflect the Turkish undergraduate pediatric dentistry curriculum and local clinical training environment. Minor wording modifications were made to improve clarity and ensure consistency with the terminology used at the study institution. No substantial changes were made to the underlying constructs assessed.

The original questionnaire items were reviewed and adapted to reflect the Turkish undergraduate pediatric dentistry curriculum and local clinical training environment. Minor wording modifications were made to improve clarity and ensure consistency with the terminology used at the study institution. No substantial changes were made to the underlying constructs assessed.

Content validity was evaluated by two specialists in pediatric dentistry. The adapted questionnaire was subsequently piloted with a small group of individuals in the dental field (including final‐year dental students, general dentists, and pediatric dentistry specialists; *n* = 10). The pilot testing was conducted to evaluate the clarity, comprehensibility, content relevance, and feasibility of the adapted questionnaire before implementation. Feedback obtained during the pilot phase was used to refine the wording and improve clarity.

The questionnaire consisted of two sections. The first section contained two items recording participants' demographic characteristics, including gender and age. The second section consisted of 15 items assessing students' self‐perceived confidence in performing pediatric dental procedures. The questionnaire included items related to patient examination and diagnosis, treatment planning, preventive procedures, restorative procedures, pulp therapy, stainless steel crown placement, local anesthesia administration, extraction of primary teeth, dental trauma management, behavior guidance, communication with parents, and perceived preparedness for independent clinical practice. Responses to all items were recorded using a 5‐point Likert scale ranging from 1 (“Strongly disagree”) to 5 (“Strongly agree”). The estimated completion time for the questionnaire was approximately 3–5 min. The internal consistency reliability of the adapted questionnaire was assessed using Cronbach's alpha coefficient (*α* = 0.927).

### 2.3 Statistical Analysis

The data were analyzed using IBM SPSS Statistics version 26.0 (IBM Corp., Armonk, NY, USA). The internal consistency of the questionnaire was assessed using Cronbach's alpha coefficient. Descriptive statistics, including frequencies, percentages, means, and standard deviations, were used to summarize the data. The normality of the data distribution was assessed using the Shapiro–Wilk test. To account for multiple comparisons, Holm's sequential correction was applied. As the data were not normally distributed, comparisons between female and male students were performed using the Mann–Whitney U test. Effect sizes and corresponding 95% confidence intervals were calculated for the principal gender comparisons. Statistical significance was set at *p* < 0.05.

## 3 Results

A total of 46 final‐year dental students who had completed their pediatric dentistry internship were included in the study. Thirty‐two participants (69.6%) were female and 14 (30.4%) were male. Twenty‐eight participants (60.9%) were aged 22–24 years and 18 (39.1%) were aged 25–26 years (Table 1).

**TABLE 1:** Demographic characteristics of participants.

Variables	Category	*n*	%
Gender	Female	32	69.6%
	Male	14	30.4%
Age	22–24	28	60.9%
	25–26	18	39.1%
Total		46	100.0%

The questionnaire responses indicated generally high levels of self‐perceived confidence across most pediatric dental procedures. The highest mean confidence scores were observed for fissure sealant application (4.24 ± 0.87), oral hygiene instruction and dietary advice (4.07 ± 0.95), topical fluoride application (4.02 ± 0.93), communication with the parent of a pediatric patient (4.02 ± 0.91), and primary tooth extraction (3.80 ± 0.86). The lowest mean confidence scores were recorded for stainless steel crown placement (2.46 ± 0.96), pulpotomy (2.72 ± 1.05), and preparedness to independently perform pediatric dental treatment after graduation (2.91 ± 1.03) (Table 2).

**TABLE 2:** Distribution of responses to the questionnaire items (%).

Questionnaire items	Strongly disagree	Disagree	Neutral	Agree	Strongly agree
Clinical examination and diagnosis	4.3	6.5	32.6	47.8	8.7
Treatment planning	4.3	13.0	21.7	56.5	4.3
Fissure sealant application	4.3	0.0	2.2	54.3	39.1
Topical fluoride application	4.3	0.0	15.2	50.0	30.4
Oral hygiene and dietary advice	6.5	0.0	2.2	63.0	28.3
Cavity preparation in primary teeth	6.5	2.2	21.7	60.9	8.7
Stainless steel crown placement	17.4	34.8	32.6	15.2	0.0
Pulpotomy	15.2	23.9	37.0	21.7	2.2
Local anesthesia administration	4.3	2.2	32.6	47.8	13.0
Primary tooth extraction	4.3	0.0	21.7	58.7	15.2
Management of uncooperative children	6.5	10.9	37.0	39.1	6.5
Dental trauma management	6.5	13.0	26.1	43.5	10.9
Preparedness for independent pediatric practice	13.0	13.0	47.8	21.7	4.3
Communication with parents	4.3	2.2	6.5	60.9	26.1
Increased confidence following the pediatric dentistry internship	4.3	4.3	10.9	56.5	23.9

Exploratory analyses comparing female and male students were performed using the Mann–Whitney U test. Initial analyses identified statistically significant gender differences for clinical examination and diagnosis (*p* = 0.018), stainless steel crown placement (*p* = 0.004), and pulpotomy (*p* = 0.008), with male students reporting higher self‐perceived confidence for these procedures. The corresponding effect sizes were moderate: clinical examination and diagnosis (*r* = 0.411, 95% CI: 0.105–0.683), stainless steel crown placement (*r* = 0.516, 95% CI: 0.194–0.790), and pulpotomy (*r* = 0.480, 95% CI: 0.183–0.732). However, none of these differences remained statistically significant after adjustment for multiple comparisons (Table 3).

**TABLE 3:** Comparison of questionnaire responses according to gender (Mann–Whitney U test).

Questionnaire items	Female Mean ± SD	Male Mean ± SD	U	*p*	Holm‐adjusted *p* ^a^
Clinical examination and diagnosis	3.31 ± 0.93	3.93 ± 0.73	132.0	0.018	0.234
Treatment planning	3.34 ± 1.00	3.64 ± 0.74	182.0	0.270	1.000
Fissure sealant application	4.19 ± 0.97	4.36 ± 0.63	213.0	0.777	1.000
Topical fluoride application	3.91 ± 1.03	4.29 ± 0.61	181.5	0.275	1.000
Oral hygiene and dietary advice	3.94 ± 1.08	4.36 ± 0.50	182.0	0.245	1.000
Cavity preparation in primary teeth	3.47 ± 1.02	4.00 ± 0.55	160.0	0.083	0.996
Stainless steel crown placement	2.19 ± 0.86	3.07 ± 0.92	108.5	0.004	0.060
Pulpotomy	2.44 ± 1.01	3.36 ± 0.84	116.5	0.008	0.112
Local anesthesia administration	3.50 ± 0.95	3.93 ± 0.73	172.0	0.183	1.000
Primary tooth extraction	3.69 ± 0.93	4.07 ± 0.62	175.0	0.191	1.000
Management of uncooperative children	3.31 ± 1.03	3.21 ± 0.89	236.0	0.771	1.000
Dental trauma management	3.22 ± 1.13	3.79 ± 0.80	160.0	0.109	1.000
Preparedness for independent pediatric practice	2.75 ± 1.02	3.29 ± 0.99	156.0	0.085	0.996
Communication with parents	3.97 ± 0.93	4.14 ± 0.86	199.0	0.501	1.000
Increased confidence following the pediatric dentistry internship	3.91 ± 0.96	3.93 ± 1.00	217.5	0.873	1.000

^a^ Statistical significance was determined based on the Holm‐adjusted *p* values (*p* < 0.05). No gender comparison remained statistically significant after Holm correction.

## 4 Discussion

The present study evaluated the self‐perceived confidence of final‐year dental students following completion of their pediatric dentistry internship. Overall, students reported higher confidence in preventive and routine clinical procedures, whereas lower confidence was observed for more technically demanding procedures, particularly stainless steel crown placement and pulpotomy. These findings may help identify areas for further review within undergraduate pediatric dentistry curricula. However, the reasons underlying the observed differences in self‐perceived confidence cannot be determined from the present study.

Dental education aims to equip students with the clinical knowledge, technical skills, and professional attitudes required for independent practice. A key component of this process is the pediatric dentistry internship, which provides direct clinical exposure to child patients and opportunities to perform fundamental pediatric dental procedures under supervision [8, 10]. Previous studies have highlighted the important role of pediatric dentistry education in promoting students' self‐efficacy and self‐confidence, particularly in communication with pediatric patients and the delivery of clinical care [7, 19]. Furthermore, practical experience in behavior management, communication, and age‐appropriate treatment planning has been shown to enhance students' preparedness for independent clinical practice after graduation [20, 21].

Lower self‐perceived confidence was observed for stainless steel crown placement and pulpotomy, which were among the most technically demanding procedures included in the questionnaire. These findings may identify areas for further review within undergraduate pediatric dentistry curricula. However, as the present study did not assess students' procedural exposure, clinical experience, or the number of procedures performed, no conclusions can be drawn regarding the underlying reasons for the lower confidence levels. Future studies incorporating objective measures of clinical experience are needed to better understand the factors influencing students' self‐perceived confidence in these procedures.

Previous studies have evaluated dental students' self‐perceived confidence across different dental disciplines, including general dentistry, prosthodontics, oral surgery, and pediatric dentistry [7, 13, 18]. Despite differences in study design and assessment methods, these studies consistently demonstrate that self‐perceived confidence is an important educational outcome that develops through clinical experience and varies according to procedural complexity [7, 18, 22, 23]. Consistent with this evidence, the present study provides an updated evaluation of self‐perceived confidence among final‐year dental students following pediatric dentistry training while adopting a more cautious interpretation of subgroup findings.

The gender‐related findings should be interpreted as exploratory because of the limited number of male participants and the multiple comparisons performed. Although the initial analyses suggested higher self‐perceived confidence among male students for clinical examination and diagnosis, stainless steel crown placement, and pulpotomy, these findings were no longer statistically significant after adjustment for multiple comparisons and should not be generalized without confirmation in larger studies. Previous studies have reported inconsistent associations between gender and self‐perceived confidence across different educational settings [5, 23]. Similarly, Gudipaneni et al. [5] found that gender, together with academic performance and clinical experience, was associated with perceived clinical competence in specific domains. Collectively, these findings suggest that self‐perceived confidence is influenced by multiple educational and individual factors rather than gender alone [5, 22, 23].

Previous studies in pediatric dentistry also support the importance of clinical experience in developing students' self‐perceived confidence. Buldur et al. [15] reported that students' perceptions of pediatric dentistry changed significantly with increasing clinical experience, whereas gender had no significant influence on these perceptions [15]. Similarly, Batista et al. [7] found lower self‐perceived confidence in invasive procedures and in the management of uncooperative pediatric patients [7]. Consistent with these findings, the present study also demonstrated lower self‐perceived confidence for more technically demanding pediatric dental procedures.

A multicenter study conducted across three dental schools in the United Kingdom evaluated students' clinical experience and self‐perceived confidence in pediatric dentistry [18]. Although the level of clinical experience varied among the participating institutions, students consistently reported higher confidence in basic procedures, such as fissure sealant application and restorative treatments, and lower confidence in more technically demanding procedures, including dental trauma management and pulp therapy [18]. These findings highlight the importance of hands‐on clinical training, particularly outreach placements, in enhancing students' self‐perceived confidence and clinical experience.

Hossain et al. [13], compared the self‐efficacy of final‐year dental students in pediatric dentistry across urban and rural educational settings. Students from both settings reported lower confidence in complex procedures, such as pulp therapy and dental trauma management, whereas those trained in urban institutions demonstrated greater confidence in basic clinical procedures [13]. These findings suggest that the educational environment may influence students' self‐perceived confidence.

The findings of the present study also have implications for competency‐based dental education. Although students reported high levels of self‐perceived confidence in several routine pediatric dental procedures, self‐perceived confidence should not be considered synonymous with clinical competence. Competency‐based dental education requires the integration of knowledge, technical skills, professional judgment, and objective assessment to ensure safe and effective clinical practice. Therefore, self‐perceived confidence should be interpreted alongside objective measures of clinical performance, such as direct clinical observation, workplace‐based assessments, and objective structured clinical examinations (OSCEs). Objective assessment methods should complement self‐reported confidence when evaluating undergraduate clinical competence.

Additionally, the study relied exclusively on self‐reported data, capturing students' self‐perceived confidence rather than objectively measured clinical competence. The absence of standardized performance assessments precludes direct comparison between self‐perceived confidence and objectively assessed clinical competence. Although the study included the entire eligible final‐year cohort, the limited number of male participants meant that it was not statistically powered for gender‐based subgroup comparisons. Therefore, the gender‐related findings should be interpreted as exploratory. Lastly, the cross‐sectional design precludes the assessment of changes in self‐perceived confidence over time. Future multicenter longitudinal studies incorporating objective assessments of clinical performance are needed to better understand the relationship between self‐perceived confidence and clinical competence during undergraduate pediatric dentistry education.

In conclusion, final‐year dental students reported high levels of self‐perceived confidence in preventive and routine pediatric dental procedures, whereas lower confidence was observed for more technically demanding procedures, particularly stainless steel crown placement and pulpotomy. These findings may help identify procedures that warrant further curriculum evaluation using objective assessments of clinical performance.

## Author Contributions

Ceren Kaplan and Melis Ünsal Çalışkaner conceived and designed the study. Ceren Kaplan collected the data, performed the statistical analyses, interpreted the findings, and prepared the original draft of the manuscript. Melis Ünsal Çalışkaner contributed to the study methodology, supervised the research process, and critically revised the manuscript for important intellectual content. Both authors read and approved the final version of the manuscript.

## Funding

The authors have nothing to report.

## Ethics Statement

This study was approved by the Non‐Interventional Scientific Research Ethics Committee of Atlas University (E‐22686390‐050.99‐66 529 Approval No. 05/16; approval date: May 26, 2025). All procedures were performed in accordance with the ethical standards of the institutional research committee and with the principles of the Declaration of Helsinki.

## Conflicts of Interest

The authors declare no conflicts of interest.

## Supporting information


**Data S1:** Supporting information.

**Data S2:** STROBE Statement—checklist of items that should be included in reports of observational studies.

## Data Availability

The data that support the findings of this study are available on request from the corresponding author. The data are not publicly available due to privacy or ethical restrictions.
