# Supplementary material for: Self‐Perceived Confidence in Pediatric Dental Procedures Among Final‐Year Dental Students
Source: Int J Paediatr Dent. 2026 Sep 11;36(6):892–8. doi: 10.1111/ipd.70142 (PMC13712563; doi:10.1111/ipd.70142)
Supplement: Supplementary file 1 [file IPD-36-892-s001.pdf]

Pedodonti Alanında Öz Yeterlilik ve Özgüven Anketi

Bu anket, XXX Üniversitesi Diş Hekimliği Fakültesi 5. sınıf öğrencilerinin çocuk hasta tedavisinde kendilerini ne derece yeterli ve özgüvenli hissettiklerini değerlendirmek amacıyla hazırlanmıştır. Katılım tamamen gönüllülük esasına dayalıdır ve yanıtlar anonim olarak toplanacaktır. Anket yaklaşık 3-5 dakika sürecektir.

* Zorunlu soruyu belirtir

1. Bu araştırma XXX Üniversitesi Diş Hekimliği Fakültesi'nde yürütülecek olup, beşinci sınıf öğrencilerinin pedodonti stajı sonrası çocuk hasta tedavisine ilişkin özgüven ve yeterlilik düzeylerini ölçmeyi amaçlayan, girişimsel olmayan, anket temelli bir çalışmadır. Araştırmaya katılım gönüllülük esasına dayanmakta ve anketler tamamen anonim şekilde toplanmaktadır. *

   Katılımcılara cinsiyet, yaş ve uyruk bilgileri sorulacak; ardından pedodonti işlemlerine yönelik özgüven düzeylerini değerlendiren 15 maddelik bir Likert tipi anket uygulanacaktır. Araştırma herhangi bir risk taşımamakta, tedavi, ilaç, girişim veya biyolojik örnek alma içermemektedir. Araştırmadan elde edilecek veriler yalnızca bilimsel amaçla kullanılacak, kimlik bilgilerinizle ilişkilendirilmeyecektir.

   Araştırmanın süresi yaklaşık 1 aydır. Katılımcı sayısı yaklaşık 35-40 öğrenci olarak öngörülmektedir.

   Bu araştırmaya katılmak istemeyebilirsiniz ve bu durumda hiçbir hak kaybı yaşamayacaksınız. Araştırmaya katıldıktan sonra da dilediğiniz zaman herhangi bir gerekçe göstermeksizin araştırmadan çekilebilirsiniz. Araştırma kapsamında size herhangi bir ödeme yapılmayacak ve sizden maddi bir talepte bulunulmayacaktır. Kimliğiniz ve verdiğiniz bilgiler gizli tutulacak, sonuçlar yalnızca bilimsel analiz amacıyla anonim şekilde raporlanacaktır.

   Yalnızca bir şıkkı işaretleyin.

   - Onaylıyorum
   - Onaylamıyorum

Bölüm 1: Demografik Sorular

2. Cinsiyetiniz nedir? *

Yalnızca bir şıkkı işaretleyin.

- Kadın
- Erkek

3. Yaşınız (yalnızca yıl olarak belirtiniz) *

4. Uyruğunuz (lütfen yazınız) *

Bölüm 2: Özgüven ve Yeterlilik Soruları

Özgüven ve Yeterlilik Değerlendirmesi

5. Çocuk hastada klinik muayene ve teşhis yaparken kendimi yeterli hissediyorum. *

Yalnızca bir şıkkı işaretleyin.

- Kesinlikle katılmıyorum
- Katılmıyorum
- Kararsızım
- Katılıyorum
- Kesinlikle katılıyorum

6. Çocuk hastaya yönelik tedavi planlaması yaparken özgüven sahibiyim. *

Yalnızca bir şıkkı işaretleyin.

- Kesinlikle katılmıyorum
- Katılmıyorum
- Kararsızım
- Katılıyorum
- Kesinlikle katılıyorum

7. Fissür örtücü uygulamasını uygun teknikle gerçekleştirebilirim. *

Yalnızca bir şıkkı işaretleyin.

- Kesinlikle katılmıyorum
- Katılmıyorum
- Kararsızım
- Katılıyorum
- Kesinlikle katılıyorum

8. Topikal florid uygulaması konusunda kendimi yeterli hissediyorum. *

Yalnızca bir şıkkı işaretleyin.

- Kesinlikle katılmıyorum
- Katılmıyorum
- Kararsızım
- Katılıyorum
- Kesinlikle katılıyorum

9. Ağız hijyeni eğitimi ve diyet önerilerini aktarma konusunda özgüvenliyim. *

Yalnızca bir şıkkı işaretleyin.

- Kesinlikle katılmıyorum
- Katılmıyorum
- Kararsızım
- Katılıyorum
- Kesinlikle katılıyorum

10. Süt dişlerine kavite preparasyonu yaparken yeterli hissediyorum. *

Yalnızca bir şıkkı işaretleyin.

- Kesinlikle katılmıyorum
- Katılmıyorum
- Kararsızım
- Katılıyorum
- Kesinlikle katılıyorum

11. Paslanmaz çelik kuron (SSC) uygulamasında özgüvenliyim. *

Yalnızca bir şıkkı işaretleyin.

- Kesinlikle katılmıyorum
- Katılmıyorum
- Kararsızım
- Katılıyorum
- Kesinlikle katılıyorum

12. Pulpotomi tedavisini klinik yönetimini gerçekleştirebilirim. *

Yalnızca bir şıkkı işaretleyin.

- Kesinlikle katılmıyorum
- Katılmıyorum
- Kararsızım
- Katılıyorum
- Kesinlikle katılıyorum

13. Lokal anesteziyi çocuk hastaya uygun şekilde uygulayabileceğime inanıyorum. *

Yalnızca bir şıkkı işaretleyin.

- Kesinlikle katılmıyorum
- Katılmıyorum
- Kararsızım
- Katılıyorum
- Kesinlikle katılıyorum

14. Süt dişi çekimini güvenle gerçekleştirebilirim. *

Yalnızca bir şıkkı işaretleyin.

- Kesinlikle katılmıyorum
- Katılmıyorum
- Kararsızım
- Katılıyorum
- Kesinlikle katılıyorum

15. Kooperasyon güçlüğü gösteren çocuk hastalarla başa çıkabilecek becerilere sahibim. *

Yalnızca bir şıkkı işaretleyin.

- Kesinlikle katılmıyorum
- Katılmıyorum
- Kararsızım
- Katılıyorum
- Kesinlikle katılıyorum

16. Dental travma durumlarında ilk müdahaleyi yapabilecek bilgi ve beceriye sahibim. *

Yalnızca bir şıkkı işaretleyin.

- Kesinlikle katılmıyorum
- Katılmıyorum
- Kararsızım
- Katılıyorum
- Kesinlikle katılıyorum

17. Mezuniyet sonrasında çocuk hasta tedavisini baştan sona uygulamaya hazırım. *

Yalnızca bir şıkkı işaretleyin.

- Kesinlikle katılmıyorum
- Katılmıyorum
- Kararsızım
- Katılıyorum
- Kesinlikle katılıyorum

18. Çocuk hastanın ebeveyniyle etkili iletişim kurabileceğime inanıyorum. *

Yalnızca bir şıkkı işaretleyin.

- Kesinlikle katılmıyorum
- Katılmıyorum
- Kararsızım
- Katılıyorum
- Kesinlikle katılıyorum

19. Pedodonti stajım, çocuk hastalarla çalışma konusundaki özgüvenimi artırdı. *

Yalnızca bir şıkkı işaretleyin.

- Kesinlikle katılmıyorum
- Katılmıyorum
- Kararsızım
- Katılıyorum
- Kesinlikle katılıyorum

Bu içerik Google tarafından oluşturulmamış veya onaylanmamıştır.

Google Formlar
